# Functional Connectivity of Anterior Insula Predicts Recovery of Patients With Disorders of Consciousness

**DOI:** 10.3389/fneur.2018.01024

**Published:** 2018-11-30

**Authors:** Li Zhang, Lunjie Luo, Zhen Zhou, Kaibin Xu, Lijuan Zhang, Xiaoyan Liu, Xufei Tan, Jie Zhang, Xiangming Ye, Jian Gao, Benyan Luo

**Affiliations:** ^1^Department of Rehabilitation Medicine, Zhejiang Provincial People's Hospital, People's Hospital of Hangzhou Medical College, Hangzhou, China; ^2^Department of Rehabilitation Medicine, The First Affiliated Hospital, School of Medicine, Zhejiang University, Hangzhou, China; ^3^College of Computer Science and Technology, Zhejiang University, Hangzhou, China; ^4^Brainnetome Center, Institute of Automation, Chinese Academy of Science, Beijing, China; ^5^Department of Rehabilitation, Hangzhou Hospital of Zhejiang CAPR, Hangzhou, China; ^6^Department of Neurology and Brain Medical Centre, The First Affiliated Hospital, College of Medicine, Zhejiang University, Hangzhou, China

**Keywords:** insula, disorders of consciousness, resting-state functional magnetic resonance imaging, functional connectivity, inferior parietal lobule

## Abstract

**Background:** We hypothesize that the anterior insula is important for maintenance of awareness. Here, we explored the functional connectivity alterations of the anterior insula with changes in the consciousness level or over time in patients with disorders of consciousness (DOC) and determined potential correlation with clinical outcomes.

**Methods:** We examined 20 participants (9 patients with DOC and 11 healthy controls). Each patient underwent resting-state functional magnetic resonance imaging (rs-fMRI) and a standardized Coma Recovery Scale-Revised (CRS-R) assessment on the same day. We categorized the patients according to the prognosis: those who emerged from a minimally conscious state (recovery group, *n* = 4) and those who remained in the unconscious state (unrecovery group, *n* = 5). Two rs-fMRI scans were obtained from all patients, and the second scan of patients in the recovery group was obtained after they regained consciousness. We performed seed-based fMRI analysis and selected the left ventral agranular insula (vAI) and dorsal agranular insula (dAI) as the regions of interest. Correlations with CRS-R were determined with the Spearman's correlation coefficient.

**Results:** Compared with healthy controls, the functional connectivity between dAI and gyrus rectus of patients who recovered was significantly increased (*p* < 0.001, cluster-wise family-wise error rate [FWER] < 0.05). The second rs-fMRI scan of patients who remained with DOC showed a significant decreased functional connectivity between the dAI to contralateral insula, pallidum, bilateral inferior parietal lobule (IPL), precentral gyrus, and middle cingulate cortex (*p* < 0.001, cluster-wise FWER < 0.05) as well as the functional connectivity between vAI to caudate and cingulum contrast to controls (*p* < 0.001, cluster-wise FWER < 0.05). Finally, the functional connectivity strength of dAI-temporal pole (Spearman *r* = 0.491, *p* < 0.05) and dAI-IPL (Spearman *r* = 0.579, *p* < 0.05) were positively correlated with CRS-R scores in all DOC patients. The connectivity of dAI-IPL was also positively correlated with clinical scores in the recovery group (Spearman *r* = 0.807, *p* < 0.05).

**Conclusions:** Our findings indicate that the recovery of consciousness is associated with an increased connectivity of the dAI to IPL and temporal pole. This possibly highlights the role of the insula in human consciousness. Moreover, longitudinal variations in dAI-IPL and dAI-temporal pole connectivity may be potential hallmarks in the outcome prediction of DOC patients.

## Introduction

The mortality after coma has decreased because of improvements in the management of emergency medicine, as a result, the number of patients diagnosed with unresponsive wakefulness syndrome/vegetative state (UWS/VS) (1) and minimally conscious state (MCS) (2) is rapidly increasing. Compared with some studies on the differential diagnosis of UWS/VS and MCS (3, 4), in our opinion, prognosis evaluation and therapeutic approaches of patients with disorders of consciousness (DOC) may be more relevant. Recently, functional neuroimaging studies have markedly contributed to the understanding of DOC, mainly showing that the default mode network (DMN) is closely related to the differential diagnosis and occurrence of DOC (5–7). The posterior cingulate cortex (PCC) (8, 9) and medial prefrontal cortex (MPFC) (10) are discovered to possibly predict outcomes in DOC patients. However, despite considerable progress in this field, the recovery mechanism of DOC remains partly elusive and an acknowledged biomarker for prognosis has not yet been found. Moreover, previous functional imaging studies have mostly included only a single scan (8, 9, 11, 12). To the best of our knowledge, rarely any resting-state functional magnetic resonance imaging (rs-fMRI) studies has obtained two longitudinal scans before and after patients regained consciousness to determine functional connectivity changes.

The insular cortex, a heterogeneous brain region which used to be considered to be connected with somatic and visceral sensory as well as motor processes (13), has recently been reported to be involved in memory, attention, language, affect, interoception, pain, decision making, sudden insight and so on (14–16). In brief, all human sensations and activities are in a way associated with the insula. Chen et al. discovered that the total time awake is reduced while total sleeping time is increased in rats with injury to the frontal insular lobe (17). Functional imaging studies have reported that insular functional connectivity changes with propofol anesthesia (18) or shamans' trance (19). Meanwhile, it is reported that the electrical disruption of the anterior insula (AI) can impair conscious awareness (20). However, although the aforementioned studies indicate a close relationship between the insula and consciousness, the actual functions of the insula in DOC remains largely unknown. In addition, although a series of studies have verified that the dorsal anterior insula (dAI) probably has more fundamental value than other parts of insula (21–23), recent studies on DOC have suggested that left ventral anterior insula (vAI) is involved unintentionally (8, 12). The insular subregion that is more relevant to DOC remains unclear. Hence, a more precise and detailed look at its function in the occurrence and outcome of DOC is warranted.

The present study searched for alterations in the functional connectivity of the anterior insula as regaining consciousness of recovery DOC patients or over time of unrecovery patients. We hypothesized that, the functional connectivity of the anterior insular would be proportional to clinical assessment scores and help to explain the mechanisms underlying the maintenance of consciousness.

## Methods

### Subjects

This study included 9 DOC patients (two MCS, seven UWS; five males; mean age = 36.8 ± 14.4 years; age range 14–54 years) who were referred to the Department of Rehabilitation in the Hangzhou Hospital of Zhejiang(CAPR), Hangzhou, China. Exclusion criteria were as follows: neuroimaging examination < 28 days since brain insult and sedation drugs received for 1 month before enrollment. Patients were categorized into two subgroups based on whether the consciousness regained or not in 18 months from the brain insult: (1) those who emerged from minimally conscious state (recovery group), (2) those who remained with DOC (unrecovery group). There were 11 normal control cases (five males, median age = 55 ± 35 years. None of the controls had any neurological or psychiatric disorders. For the detailed demographic and clinical information of the DOC patients and controls is presented in Table 1.

**Table 1:** Demographic and clinical information for subjects.

**Subjects**	**Age range(y)**	**Etiology**	**Time to MRI (d)**	**CRS-R score**	**CRS-R subscore**
Patient 1	26–30	TBI	102	7 (MCS)	113002
			311	23 (EMCS)	456323
Patient 2	16–20	TBI	28	7 (MCS)	113002
			83	23 (EMCS)	456323
Patient 3	11–15	TBI	72	1 (UWS)	001000
			404	22 (EMCS)	456313
Patient 4	26–30	TBI	47	2 (UWS)	000002
			92	14 (MCS)	345002
			450	23 (EMCS)	456323
Patient 5	46–50	Anoxia	28	4 (UWS)	011002
			117	7 (UWS)	112102
Patient 6	46–50	TBI	81	6 (UWS)	112002
			173	7 (UWS)	112102
Patient 7	46–50	TBI	32	5 (UWS)	102002
			140	6 (UWS)	112002
Patient 8	41–45	Anoxia	28	4 (UWS)	101002
			54	4 (UWS)	101002
Patient 9	51–55	TBI	67	5 (UWS)	111002
			93	11 (MCS)	233102
Control 1	26–30	/	/	/	/
Control 2	21–25	/	/	/	/
Control 3	26–30	/	/	/	/
Control 4	16–20	/	/	/	/
Control 5	51–55	/	/	/	/
Control 6	61–65	/	/	/	/
Control 7	56–60	/	/	/	/
Control 8	66–70	/	/	/	/
Control 9	51–55	/	/	/	/
Control 10	61–65	/	/	/	/
Control 11	56–60	/	/	/	/

*CRS-R, coma recovery scale-revised; MCS, minimally conscious state; UWS, unresponsive wakefulness syndrome; EMCS, emergence from minimally conscious state; TBI, traumatic brain injury*.

There were no contradictions to MRI scan among the DOC patients or controls. For the controls, a rs-fMRI scan was performed immediately after enrollment. For the patients, rs-fMRI scans were performed as soon as their conditions were stabilized. UWS, MCS and emergence from MCS (EMCS) were diagnosed according to The Coma Recovery Scale-Revised (CRS-R) (24). The CRS-R assessments were performed by an experienced physician of rehabilitation on the same day as the MRI scanning. The scale comprises six subscales including auditory function, visual function, motor function, verbal function, communication, and arousal components. Following the recovery of consciousness, follow-up rs-fMRI and reassessment of the CRS-R were performed. For patients who had not yet recovered consciousness, fMRI was performed on a chosen date. The first and second rs-fMRI scans were labeled as T1 and T2, respectively.

All healthy participants and the legally authorized representatives of the patients received a complete description of the study and provided written informed consent. This study was approved by the Ethics Committee of the First Affiliated Hospital, School of Medicine, Zhejiang University.

### Data acquisition

All MRI scans were obtained with the same Magnetom Essenza 1.5 Tesla scanner (Siemens, Germany). A highresolution T1-weighted sequence for localization was acquired (repetition time [TR] = 2,000 ms, echo time [TE] = 5.18 ms, flip angle [FA] = 15°, acquisition matrix = 256 × 256, field of view [FOV] = 240 mm × 240 mm, thickness = 1.0 mm, gap = 0.5 mm). T2-weighted functional images were obtained with a gradient-echo echo-planar imaging sequence (TR = 2000 ms, TE = 40 ms; FA = 90°, acquisition matrix = 64 × 64, FOV = 240 mm × 240 mm, thickness = 4.0 mm, gap = 0 mm). This acquisition sequence generated 240 volumes in 8 min.

### Data preprocessing

We preprocessed the rs-fMRI data using the MATLAB-based resting state fMRI processing toolkit BRANT (http://brant.brainnetome.org). The first 10 volumes of each functional time series were discarded, 230 volumes remained. Preprocessing steps included (1) slice-time correction, (2) rigid-body motion correction using realign, (3) coregistration of T1 structural data to resting fMRI data, (4)normalization to 3 × 3 × 3 mm^3^ cubic voxels, (5) removal of confounding factors via linear regression (i.e., the 24 motion parameters, and the mean time series of all voxels within the white matter and cerebrospinal fluid) without global signal regression, (6) temporal filtering (0.01–0.08 Hz), and (7) smoothing with a 6-mm full width and half maximum kernel (25, 26). Key exclusion criteria were large focal brain damage, motion parameters of more than 3 mm in translation and 3 degrees in rotation.

### Defining regions of interest

**Figure 1:**
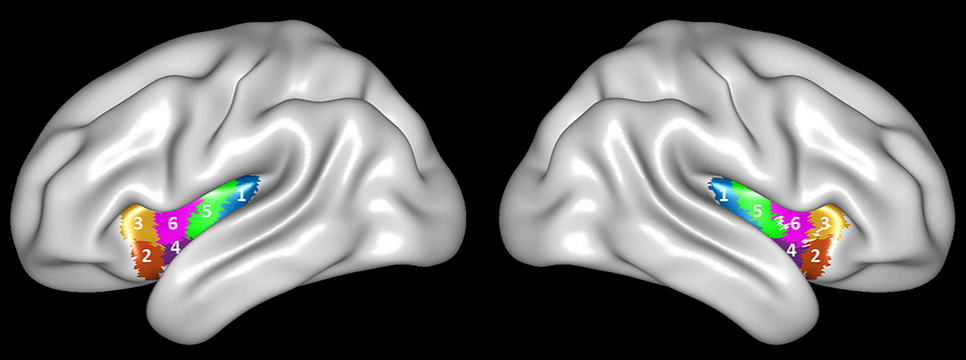
Brainnetome Atlas of the bilateral insular cortex. Six subregions (INS-1 to INS-6) were identified in each side of the insular cortex (27).

According to the Human Brainnetome Atlas (http://atlas.brainnetome.org), the insula was divided into six sub-regions, covering INS-1 (G, hypergranular insula), INS-2 (vIa, ventral agranular insula), INS-3 (dIa, dorsal agranular insula), INS-4 (vId/vIg, ventral dysgranular and granular insula), INS-5 (dIg, dorsal granular insula), and INS-6 (dId, dorsal dysgranular insula) of the insula at both sides (Figure 1). We selected the left INS-2 (equivalent to ventral anterior insula) and INS-3 (equivalent to dorsal anterior insula) as the regions of interest (ROI).

### Functional connectivity analyses

To examine resting-state functional connectivity, we used a seed-based approach. A voxel-wise functional connectivity analysis was performed separately for each ROI. Moreover, the functional connectivity strength was acquired by computing Pearson's correlation coefficient between the average time series for one seed and each voxel in the brain. The mean time series for all voxels within the ROI were used as the seed reference time series. For further statistical analysis, the correlation coefficients were transformed into Z-values using the Fisher r-to-z transformation (26).

### Statistical analysis

For the comparison of fMRI resting state functional connectivity, we used SnPM for non-parametric two sample tests (permutation test) and cluster-extent multiple comparison correction (voxel-wise *P* < 0.001, cluster-wise FWER < 0.05). The effects of age and sex were adjusted. The analysis was conducted between two scans obtained at different timepoints from the recovery group and unrecovery group, and to the control group respectively.

SPSS 23.0 was used for statistic analyses. The Spearman's rank correlation approach was adopted to analyze the correlation between the functional connectivity strength and the CRS-R scores. *p* ≤ 0.05 was considered statistically significant.

## Results

### Characteristics of the subjects

Among the 9 DOC cases, 4 subjects (patients 1–4) recovered consciousness, indicating that they achieved EMCS. The other 5 cases (patients 5–9) were followed up for 1.5 years without recovery of consciousness, and their Glasgow Coma Scale scores remained at 2 points. No significant differences were observed between the recovery and unrecovery groups in terms of sex (*p* > 0.05), time course of the first rs-fMRI scan (*p* > 0.05), and CRS-R scores at T1 (*p* > 0.05). The age of subjects in the control and unrecovery groups were not significantly different (*p* > 0.05) (Table 2).

**Table 2:** Summarized demographic and clinical information for subjects.

	**Rec (*N* = 4)**	**Unrec (*N* = 5)**	**Con (*N* = 11)**	***P-*value (Rec vs. Unrec)**	***P-*value (Rec vs. Con)**	***P-*value (Unrec vs. Con)**
Age (y)	22.8 ± 7.5	48.0 ± 4.3	55 ± 35	< 0.001	0.077	0.427
Gender	3 M, 1F	2 M, 3F	5 M, 6 F	0.524	0.569	1.000
Time to MRI at T1(d)	62.3 ± 32.1	51.2 ± 23.4	/	0.567	/	/
CRS-R scores at T1	4.3 ± 3.2	4.8 ± 0.8	/	1.000	/	/
Time to MRI at T2(d)	312.0 ± 163.2	115.4 ± 45.3	/	0.035	/	/
CRS-R scores at T2	23.0 ± 1.0	7.0 ± 2.6	/	0.016	/	/

*Rec, recovery group; Unrec, unrecovery group; Con, control group*.

### Analysis of the anterior insular functional connectivity

Because of the limited sample size of the recovery and unrecovery groups, comparisons between T1 and T2 rs-fMRI in both groups failed to pass the permutation test. However, we still found some intriguing functional connectivity changes between the patients and controls.

**Figure 2:**
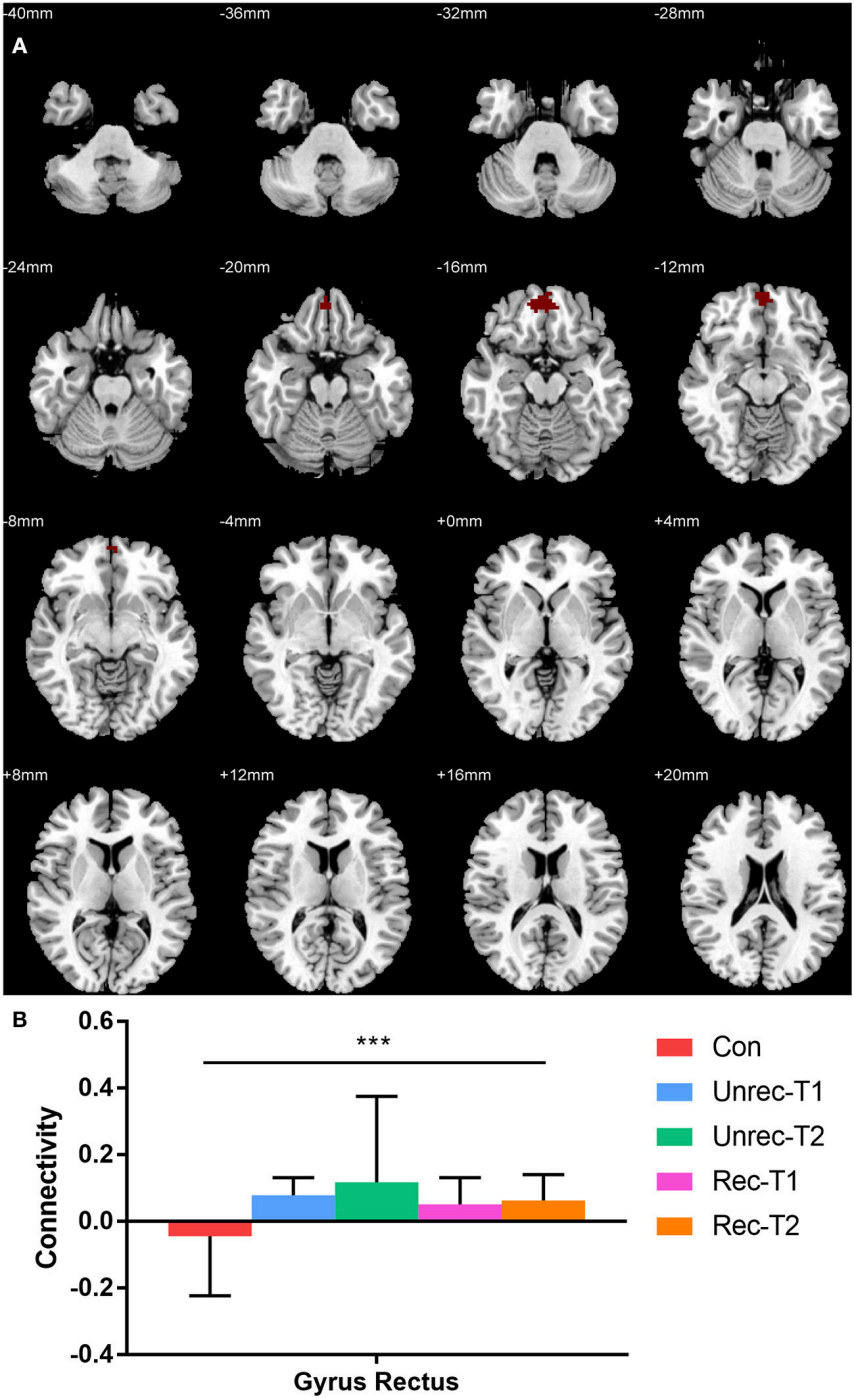
Increased functional connectivity with dorsal anterior insula (dAI) in DOC. **(A)** shows the regions with increased functional connectivity to the dAI in the recovery group at T2. **(B)** shows the Z values of the corresponding functional connectivity in the control group, unrecovery group at T1/T2, and recovery group at T1/T2 (***p < 0.001, cluster-wise FWER < 0.05). Error bars represent standard deviation.

**Figure 3:**
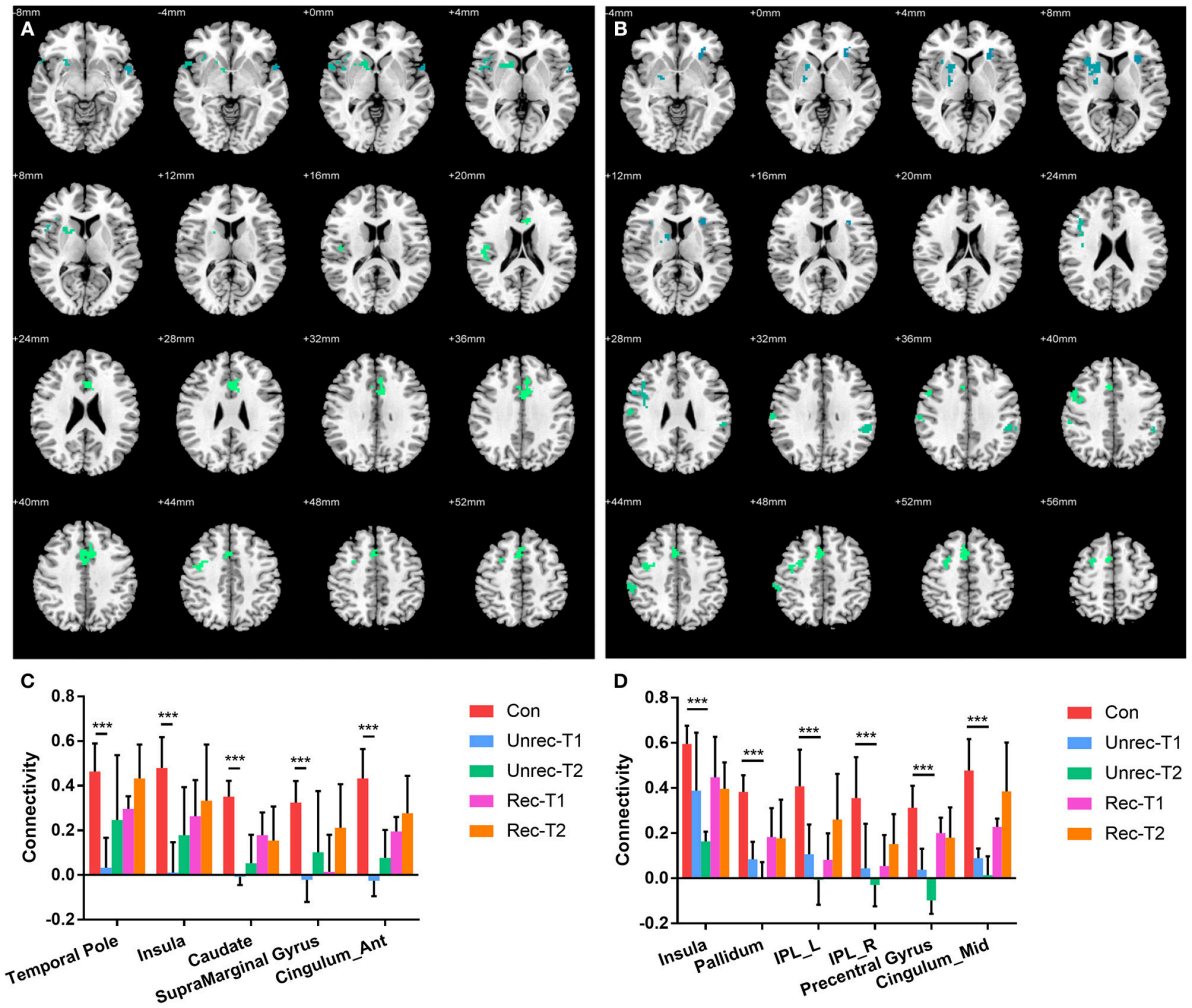
Decreased functional connectivity with dorsal anterior insula (dAI) in DOC. **(A)** and **(B)** show the regions with decreased functional connectivity to the dAI in the unrecovery group at T1 and T2, respectively. **(C,D)** display the Z values of the corresponding functional connectivity in the control group, unrecovery group at T1/T2, and recovery group at T1/T2 (***p < 0.001, cluster-wise FWER < 0.05). Error bars represent standard deviation.

Our observation was consistent with our hypothesis that both the dAI and vAI showed important functional connectivity changes in DOC patients (coordinates of regions, Tables 3-1,3-2). Considering the dAI as the ROI, we found functional connectivity alterations in both the unrecovery and recovery groups. The functional connectivity strength between the dAI and gyrus rectus of patients who regained their consciousness was significantly higher than that in the controls (*p* < 0.001, corrected; Figure 2). Moreover, the unrecovery group at T1 had decreased functional connectivity between the dAI and temporal pole, contralateral insula, caudate, supramarginal gyrus, and anterior cingulate cortex (ACC) (*p* < 0.001, corrected; Figures 3A,C). In addition, the functional connectivity of the dAI to contralateral insula, pallidum, bilateral inferior parietal lobule (IPL), precentral gyrus, and middle cingulate cortex of the unrecovery group at T2 was also decreased compared with the controls (*p* < 0.001, corrected) (Figures 3B,D). No significant difference was observed in Z values between the recovery and unrecovery group at T1 or T2.

**Table 3-1:** Coordinates of regions with changed functional connectivity to the dAI (vs. Control).

					**MNI coordinates**		
**Group**	**Time of fMRI**	**Change trend**	**Brain region**	**Location**	**X**	**Y**	**Z**
Rec	T2	Increased	Gyrus rectus	L	0	48	−21
Unrec	T1	Decreased	Temporal pole	L	48	18	−18
	T1	Decreased	Insula	R	24	12	−12
	T1	Decreased	Caudate	R	9	3	−3
	T1	Decreased	Supramarginal gyrus	R	45	−18	15
	T1	Decreased	Cingulum_ant	L	−6	21	18
Unrec	T2	Decreased	Insula	L	−27	24	−6
	T2	Decreased	Pallidum	R	28	−6	−3
	T2	Decreased	Inferior parietal lobule	L	−60	−33	27
	T2	Decreased	Inferior parietal lobule	R	60	−18	27
	T2	Decreased	Precentral gyrus	R	51	6	36
	T2	Decreased	Cingulum_mid	R	6	15	36

**Table 3-2:** Coordinates of regions with changed functional connectivity to the vAI (vs. Control).

					**MNI coordinates**		
**Group**	**Time of fMRI**	**Change trend**	**Brain region**	**Location**	**X**	**Y**	**Z**
Unrec	T1	Decreased	Putamen	R	27	0	0
Unrec	T2	Decreased	Caudate	R	24	9	3
	T2	Decreased	Cingulum	R	6	12	24

*Rec, recovery group; Unrec, unrecovery group; Con, control group*.

**Figure 4:**
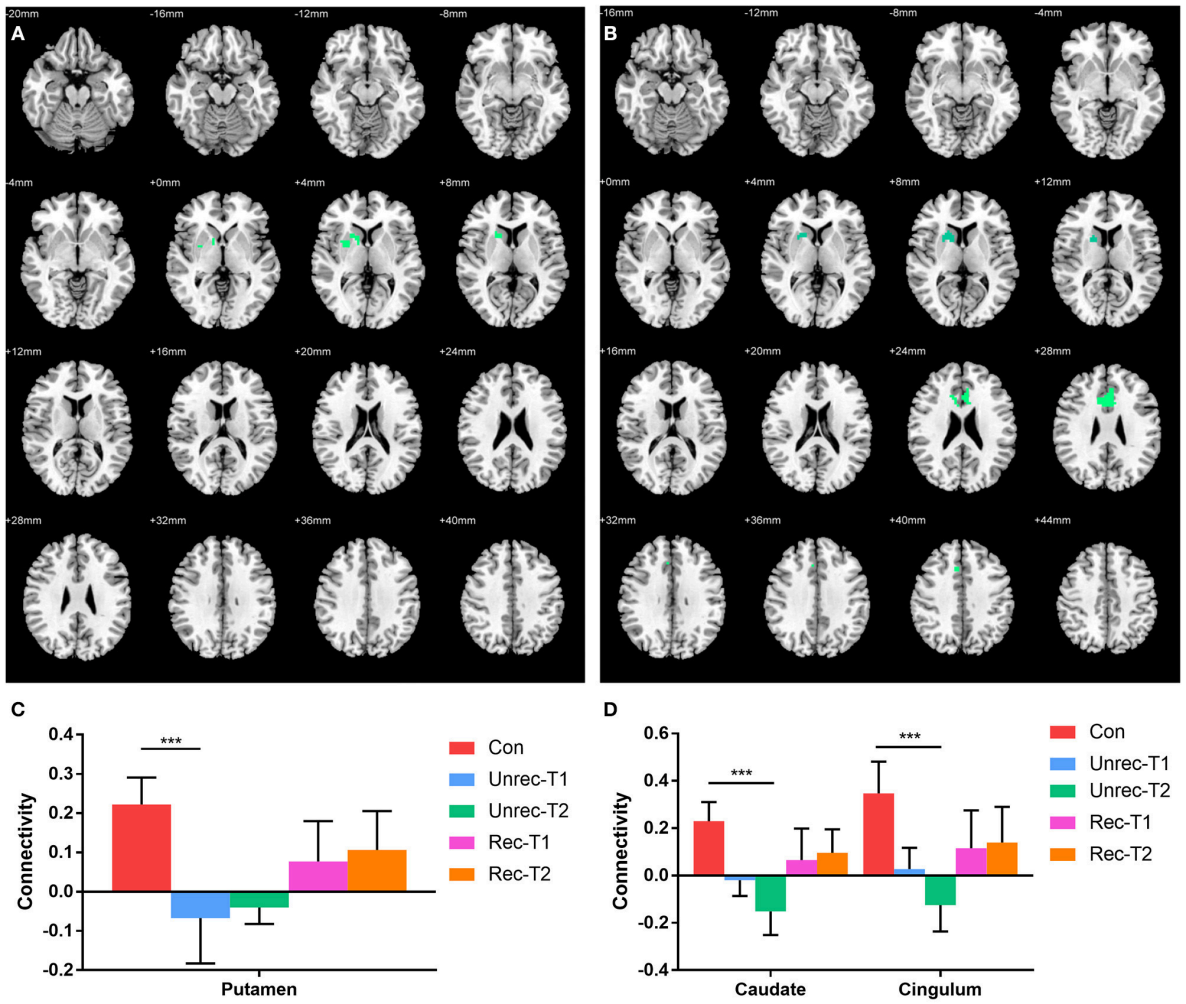
Decreased functional connectivity with ventral anterior insula (vAI) in DOC. **(A,B)** show the regions with decreased functional connectivity to the vAI in the unrecovery group at T1 and T2, respectively. **(C,D)** display the Z values of the corresponding functional connectivity in the control group, unrecovery group at T1/T2, and recovery group at T1/T2 (***p < 0.001, cluster-wise FWER < 0.05). Error bars represent standard deviation.

In Figure 4, we report the group-level results of seed-based connectivity analyses obtained when the vAI was considered as the seed. Although we compared two scans obtained from each patient group to those obtained from the controls, only the unrecovery group revealed differences. The functional connectivity between the vAI and putamen in the unrecovery group at the first rs-fMRI scan (T1) was significantly decreased compared with that in the control group (*p* < 0.001, corrected; Figures 4A,C). In addition, the connectivity from the vAI to caudate or cingulum of the unrecovery patients at the second rs-fMRI scan (T2) was significantly disrupted (*p* < 0.001, corrected; Figures 4B,D). No significant difference was observed in Z values between the recovery and unrecovery groups at T1 or T2.

### Correlation between the functional connectivity and clinical assessments

**Figure 5:**
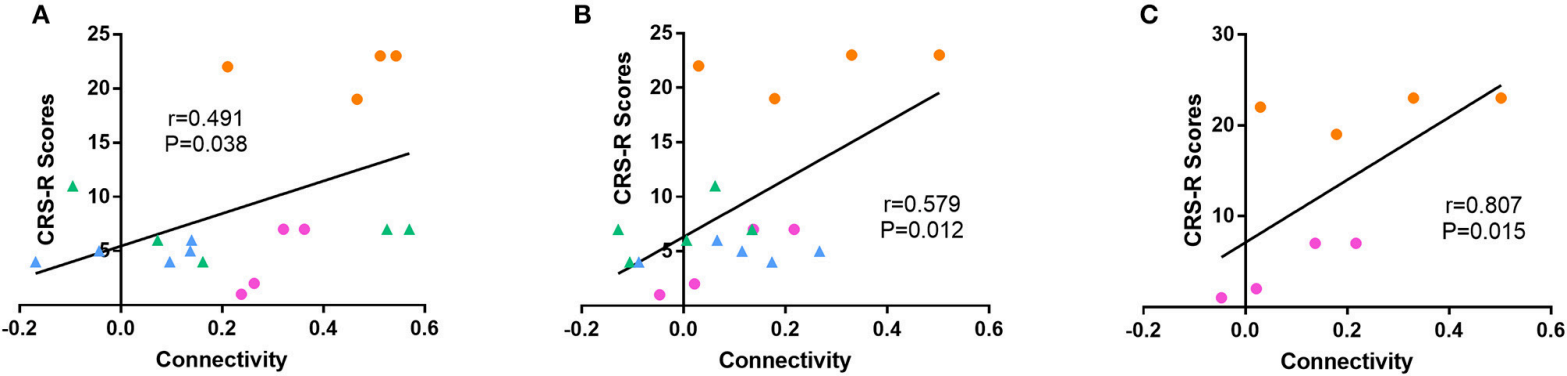
Correlation between functional connectivity and the CRS-R score. Linear analyses showed a significant positive correlation of **(A)** dAI-temporal pole (Spearman *r* = 0.491, *p* < 0.05) and **(B)** dAI-IPL_L (Spearman *r* = 0.579, *p* < 0.05) functional connectivity in all patients to the CRS-R score. **(C)** Linear analyses showed a significant positive correlation of dAI-IPL_L in the recovery group (Spearman *r* = 0.807, *p* < 0.05). (blue = unrecovery group at T1, green = unrecovery at T2, pink = recovery group at T1, and orange = recovery group at T2).

Among all the aforementioned connectivity, the correlation of functional connectivity in patients with CRS-R scores were examined. The result of linear analysis demonstrated a significant positive correlation in dAI-temporal pole (Spearman *r* = 0.491, *p* = 0.038) and dAI-IPL_L (Spearman *r* = 0.579, *p* = 0.012) (Figures 5A,B). Complementary analysis of the correlation of dAI-temporal pole and dAI-IPL_L in the recovery group showed that only connectivity of dAI-IPL_L had a significant positive correlation with the clinical scores (Spearman *r* = 0.807,*p* = 0.015) (Figure 5C).

## Discussion

Our findings mainly revealed that the functional connectivity strength between the dAI and gyrus rectus of the recovery patients was higher than that of the controls. In addition, compared with controls, many brain regions in DOC patients of the unrecovery group showed reduced functional connectivity with the dAI and vAI, such as the temporal pole, caudate, supramarginal gyrus, and cingulum at baseline, pallidum, IPL, precentral gyrus, and middle cingulate in the follow-up period. Notably, the connectivity between the dAI and IPL as well as that between the dAI and temporal pole showed a significant positive correlation to the clinical scores.

Although we could not observe a direct functional connectivity change in patients with DOC as consciousness regaining, we believe that the present findings are significant because they reflex the functional connectivity change indirectly compared with the controls. In our opinion, results from the second rs-fMRI scan were more relevant to the study of outcomes of DOC patients.

The insula not only integrates various sensations, including smell, taste, touch, pain and interoception (15), (22), but also participates in the connectivity of the salience network (SN) (28), the limbic system (6), and the external awareness network (29). Previous studies have reported that the dAI is important for the integration of insular subregions (14, 22), therefore, it is a vital regulatory hub for the brain network (21, 23). The dAI is functionally connected to frontal areas, such as the ACC and dorsolateral prefrontal cortex (DLPFC) (21, 22). The vAI is chiefly functionally connected to the limbic system, nucleus accumbens, superior temporal sulcus, and orbitofrontal cortex. The vAI mainly associated with emotions and chemical sensation (14, 21, 22, 30). Qin et al. reported that in UWS patients, the intensity of connectivity of the supragenual anterior cingulate cortex (SACC) to the left anterior insula is significantly reduced compared with that in MCS patients (8). Fischer et al. discovered that the damage to connectivity is more significant between the vAI and pregenual anterior cingulate cortex (pACC) than in the default network and motor network in DOC patients (12). In that study, the authors also mentioned that the ventral anterior insula, not the dorsal anterior insula or the right insula, was associated with DOC. Growing evidence has showed a more important function of the left hemisphere related to consciousness (20, 31, 32). Therefore, we selected the left dAI and vAI as the ROIs in our study. With respect to the current results, we presume that both dAI and vAI might be related to the recovery mechanism of DOC.

In our study, except for the functional connectivity between dAI and gyrus rectus, other functional connectivity of AI were all reduced compared to controls. Some previous studies have also shown a decrease in AI functional connectivity in DOC patients (8, 12), whereas other studies have indicated hyperconnectivity (6) or hyperactivity (29) of the insula in these patients, demonstrating an inconsistency in this area. Hyperconnectivity between the ACC and insula or the PCC and insula is more significant in UWS patients than in MCS patients, indicating that these may be self-reinforcing dysfunctional connections (6). The difference in the results may be because of differences in the illness duration, methods of analyses, and patients classification.

Here, we showed a positive correlation between increases in the intensity of functional connectivity of dAI-IPL as well as dAI-temporal pole and the improved CRS-R scores. In addition, the positive correlation between dAI-IPL and consciousness level was found in all DOC patients, particularly in the recovery group. These findings indicated that the functional connectivity of dAI-IPL might be a potential index for the prediction the restoration of the consciousness level.

Notably, part of the aforementioned regions belonged to the DMN, including the IPL and temporal pole (33, 34). The DMN can reflect the baseline state in the human brain, mainly containing the PCC, precuneus(PCU), MPFC (35). Numerous studies have demonstrated that the functional connectivity of the DMN decreases in DOC patients (11, 36–38). However, previous studies principally focused on the PCC, PCU and MPFC within the DMN, and were rarely concerned about the IPL or temporal pole. The present results demonstrated that the connectivity of IPL and temporal pole to the AI significantly decreased in patients with unsuccessful consciousness restoration over time and showed a positive correlation with the consciousness level.

Some functional connectivity of AI in the unrecovery group decreased at T2 when it demonstrated an opposite variation trend in the recovery group, (e.g., the IPL, cingulate and caudate). These regions are deserved to be focused on in the future for understanding the mechanism underlying DOC.

At present, the measures for waking up a DOC patient include various external stimuli, such as median nerve electrical stimulation and Chinese acupuncture. Considering the vital role of the insula in sensation integration (15, 22), it may become an important target in the treatment strategies to awaken DOC patients.

In this paper, we adopted the Human Brainnetome Atlas constructed by the team of Prof. Jiang of the Chinese Academy of Science (27). According to this atlas, the insula of either side is divided into six subregions, and this division is the basis for the detailed study of insular function in the recovery of DOC patients.

The study has several limitations. First, only a small number of patients regained consciousness and completed two rs-fMRI examinations. Thus, the present results must be verified in a larger sample. Second, DOC patients may be asleep while undergoing rs-fMRI examination, and this may affect the study results. In the future, electroencephalography may be used in combination with rs-fMRI. This would exclude the influence of sleep on the examination results. Third, etiological heterogeneity exists in this study, and further analyses may be made according to the cause of illness to enhance the reliability of the results.

In summary, this paper may be the first study focusing on the effects of functional connectivity alternations of insula in the mechanism of DOC. Here, we found that both dorsal AI and ventral AI might related to the recovery of consciousness. Furthermore, the intensity of functional connectivity between dAI to IPL and temporal pole showed positive correlations with consciousness level, therefore, they could be considered to be used as an valuable indicators in the predicting the outcome for DOC patients.

## Author contributions

LZ and LL contributed equally to this work. LZ, LL, XL, XT, and JZ were involved in the study design, interpretation of data and manuscript drafting. LJZ was responsible for acquisition of data. ZZ, KX were responsible for data analysis. XY, JG, and BL were responsible for the study supervision. All authors contributed to manuscript revision, read and approved the submitted version.

### Conflict of interest statement

The authors declare that the research was conducted in the absence of any commercial or financial relationships that could be construed as a potential conflict of interest.

## Abbreviations

DOC: Disorders of Consciousness
CRS-R: Coma Recovery Scale-Revised
MCS: Minimally Conscious State
VS/UWS: Vegetative State/Unresponsive Wakefulness Syndrome
EMCS: Emergence From Minimally Conscious State
rs-fMRI: Resting-State Functional Magnetic Resonance Imaging
DMN: Default Mode Network
SN: Salience Network
vAI: ventral Anterior Insula
dAI: dorsal Anterior Insula
ROI: Regions of Interest
IPL: Inferior Parietal Lobule.
